# Taste perception and muscular response: EMG based experimental evaluation

**DOI:** 10.1016/j.jobcr.2025.02.016

**Published:** 2025-03-11

**Authors:** Bhavya Rohatgi, Ramya Ramadoss, K. Nitya, Sandhya Sundar, Suganya Panneer Selvam, K. Hema Shree

**Affiliations:** a Department of Oral Biology, Saveetha Dental College and Hopsitals, Saveetha Institute of Medical and Technical Sciences, Saveetha University, Chennai, 600077, India; b Department of Oral Biology and Oral Pathology, Saveetha Dental College and Hopsitals, Saveetha Institute of Medical and Technical Sciences, Saveetha University, Chennai, 600077, India

**Keywords:** Taste perception, Electromyography, Masseter, Temporalis, Muscle synergy, Muscle symmetry, Masticatory muscles

## Abstract

**Background:**

Taste perception influences not only flavor preference but also the physiological processes of chewing and digestion. Each primary taste—sweet, sour, salty, bitter, and umami—affects specific receptors and shapes masticatory muscle activity, notably in the temporalis and masseter muscles. Limited research exists on how taste affects muscle synergy (coordinated activation) and symmetry (balanced activation) in chewing. Using surface electromyography (EMG), this study examines how different tastes impact these muscle dynamics, offering insights relevant to dentistry, nutrition, and food science.

**Methods:**

This study included five healthy participants aged 18–22 years. EMG recordings were conducted on the temporalis anterior and masseter muscles. Each participant chewed five distinct jelly formulations (sweet, sour, salty, bitter, umami) prepared with standardized flavours. The muscle activity was analyzed to determine the effects of taste on muscle synergy (right and left temporalis and masseter) and symmetry (temporalis and masseter). Paired t-tests and ANOVA were used to assess statistical significance.

**Results:**

The results revealed taste-dependent variations in muscle synergy and symmetry. Sweet and salty tastes increased muscle synergy, while bitter and umami decreased it. Minimal changes were observed in the symmetry of the temporalis muscle across taste conditions, while the symmetry of the anterior masseter showed notable variations, especially with salty and umami. However, statistical analysis indicated no significant differences in muscle synergy or symmetry between jelly and non-jelly conditions across all taste stimuli (p > 0.05).

**Conclusion:**

This study underscores the complexity of the neuromuscular response to taste perception, suggesting potential subtle influences of taste on muscle activity. Future research with a larger sample size and advanced statistical methods may further elucidate the role of taste in modulating masticatory muscle function.

## 1 Introduction

**Fig. 1:**
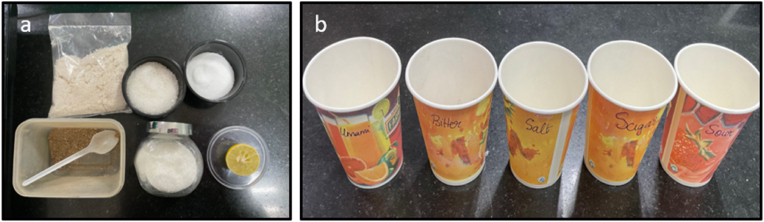
Ingredients to make the gel- Salt, Sugar and 5 different tastes-Sweet, Sour, Salty, Bitter and Umami.

The sensory experience of taste, or gustation, is crucial not only for the enjoyment of food but also for its broader physiological impacts, particularly on muscle function. The five primary taste modalities—sour, sweet, salty, bitter, and umami—each have distinct biological roles that extend beyond flavor detection. Taste receptors, especially G-protein-coupled receptors (GPCRs), are responsible for detecting these tastes and are found not only in the mouth but also in other tissues, including muscles, where they influence metabolism and muscular function. Sweet taste, for instance, regulated by GPCRs, is connected to glucose metabolism and insulin response, both of which are key to muscle endurance and performance (Laffitte et al., 2014^1^). Similarly, ion channels that mediate salt taste help maintain electrolyte balance and fluid homeostasis, critical for nerve impulse transmission and muscle contraction (Breslin 2013).^2^ These examples highlight the profound connection between taste perception and muscular activity (see Table 1, Fig. 1)

**Table 1:** Comparison of muscle synergy and symmetry in temporalis and anterior masseter: T-test results indicating non-significant differences (p > 0.05).

Variables	Mean ± SD (with jelly)	Mean ± SD (without jelly)	T-Statistic	P value
Synergy_Right	76.8 **±** 2.1	77.4 ± 1.9	0.929	0.379
Synergy_Left	77.2 **±** 2.0	77.3 ± 1.8	−0.064	0.534
Symmetry_Temporalis	77.5 **±** 2.0	77.6 ± 1.7	0.0632	0.544
Symmetry_Anterior_Masseter	77.0 **±** 2.0	78.3 ± 2.2	1.333	0.218

The role of taste in muscle function is particularly significant when considering the muscles of mastication, which include the masseter, temporalis, and pterygoid muscles. These muscles are essential for chewing and breaking down food, and understanding how different tastes influence their activity could offer valuable insights into the neuromuscular mechanisms involved in eating. Sweet tastes, for instance, may enhance muscle performance by influencing glucose metabolism, which is essential for endurance activities and prolonged muscle activity.^3^ Salt, by preserving electrolyte balance, plays a critical role in maintaining proper muscle function and contraction.^4^ Even bitter tastes, usually associated with detecting harmful substances, can modulate muscle activity by influencing nutrient absorption and energy utilization through gastrointestinal receptors.^5^^,^^6^ *This understanding could lead to improved dietary strategies that optimize chewing efficiency, digestion, and overall nutrition. By tailoring taste stimuli to improve masticatory muscle function, these findings may contribute to clinical interventions aimed at enhancing oral health, promoting efficient digestion, and addressing eating disorders.*

Taste perception also affects oral health, with variations in muscle activity potentially leading to conditions such as temporomandibular disorders (TMD) or dental issues.^7^^,^^8^ Excessive or insufficient activation of the masticatory muscles in response to different taste stimuli could lead to abnormal wear and tear on teeth, jaw pain, or muscle fatigue, affecting oral health. Understanding the impact of different tastes on muscle contraction could provide a clinical framework for treating masticatory dysfunction or TMD.^9^ For instance, therapies tailored to modulate muscle activity through dietary adjustments could enhance patient outcomes, particularly in those with swallowing disorders or impaired chewing function.^10^ *The translational value of this research lies in its potential to bridge sensory perception and motor function, providing therapeutic strategies for oral health disorders and rehabilitation programs for patients suffering from neuromuscular dysfunction. Such* an approach bridges the gap between sensory perception and motor output, contributing to broader fields such as sensory-motor integration and rehabilitation science.

Finally, the implications of taste on muscle function extend beyond the oral cavity. For example, umami taste, which detects glutamate and amino acids, has been linked to muscle protein synthesis and post-exercise recovery, making it relevant not just to mastication but to general muscle health.^6^^,^^11^ Similarly, sour taste, caused by hydrogen ions, can influence muscle contraction through its effect on body pH and acid-base balance. *These findings can be applied to sports nutrition, muscle recovery programs, and clinical settings to enhance physical performance and rehabilitation outcomes*. These insights underscore the intricate relationship between taste perception and muscle activity across the body, not just in mastication but also in other motor functions influenced by sensory stimuli. Research into how taste receptors affect muscle performance could inform future studies in neurophysiology and rehabilitation, offering new perspectives on how sensory inputs modulate motor responses across different physiological systems.

## 2 Materials and methods

### 2.1 Study participants

The study included five healthy individuals aged between 18 and 22 years, all free from systemic comorbidities. Informed consent was obtained from all participants prior to their inclusion in the study. Ethical clearance was granted in accordance with institutional guidelines.

### 2.2 Experimental design

A within-subjects experimental design was employed to evaluate pre-treatment and post-treatment changes in the muscular activity of the temporalis and masseter muscles in response to five distinct taste stimuli: sweet, sour, salty, bitter, and umami. Standardized jelly formulations, each infused with one of the five taste stimuli, were used as test samples. These jellies were prepared under controlled laboratory conditions to ensure uniform texture and flavour concentration across all samples.

### 2.3 Taste sample preparation

Five distinct jelly samples were prepared, each representing one of the five basic tastes: salty, sweet, sour, umami, and bitter. The salty jelly was flavoured with salt, while sugar was used for the sweet variety. Lemon extract was incorporated to impart a sour taste, and monosodium glutamate (MSG) provided the umami flavour. The bitter jelly was created using Kabasura Kudineer, a traditional herbal formulation known for its therapeutic properties. China grass extract was used as the base ingredient for all the jellies, ensuring uniformity in texture across the samples. Equal portions of each jelly were prepared, maintaining consistency in taste exploration.

### 2.4 Electromyographic (EMG) recording

Surface electromyography (EMG) was employed to assess the muscular activity of the temporalis anterior and masseter muscles, following standard anatomical guidelines for precise data collection. For the masseter muscle, electrodes were placed over its belly, approximately at the midpoint between the zygomatic arch and the angle of the mandible. The temporalis anterior muscle was targeted by positioning the electrodes around 2–3 cm above the zygomatic arch, aligned with the coronal suture.^5^ This careful electrode placement ensured accurate and reliable measurements of muscle activity during the assessment.

### 2.5 Baseline recording and taste stimulation procedure

EMG activity was first recorded with an empty oral cavity to establish a baseline for comparison. Following this, participants were instructed to chew randomized taste samples to prevent order effects. During the chewing process, EMG activity of the temporalis and masseter muscles was recorded for each taste stimulus. This procedure allowed for the analysis of how different tastes influenced muscle activity.

### 2.6 Muscle synergy and symmetry assessment

The synergy between the temporalis and masseter muscles was assessed by comparing EMG activity on the same side (e.g., right temporalis with right masseter, left temporalis with left masseter). Symmetry was evaluated by comparing the EMG activity between the right and left temporalis muscles, as well as between the right and left masseter muscles.

### 2.7 Data acquisition, variables, and statistical analysis

Two primary variables were analyzed: the average time to first muscle firing, representing the latency from the onset of chewing to detectable muscle activity, and the peak EMG amplitude, which measured the action potential during muscle contraction. Data were processed using specialized software, and paired t-tests along with ANOVA were employed to identify significant differences in muscle symmetry and synergy across taste stimuli, with a p-value of <0.05 considered statistically significant. Reliability of the EMG recordings was ensured through calibration before each session, and intra-rater and inter-rater reliability assessments were conducted to validate the findings.

**Fig. 2:**
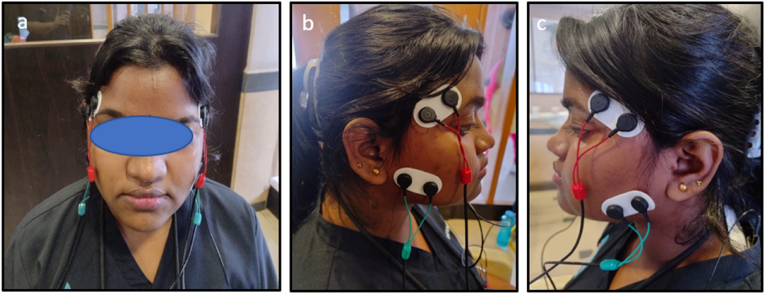
EMG Electrodes applied for Masseter [green] (Right and Left) and Temporalis Anterior [Red] (Right and Left); (b) and (c) Red electrodes for Temporalis Anterior and Green electrodes for Masseter muscle. (For interpretation of the references to colour in this figure legend, the reader is referred to the Web version of this article.)

Paired t-tests were performed to compare the pre-treatment and post-treatment EMG activity in response to different taste stimuli. Differences in muscle symmetry and synergy were analyzed with significance set at p < 0.05 (see Fig. 2).

## 3 Results

**Fig. 3:**
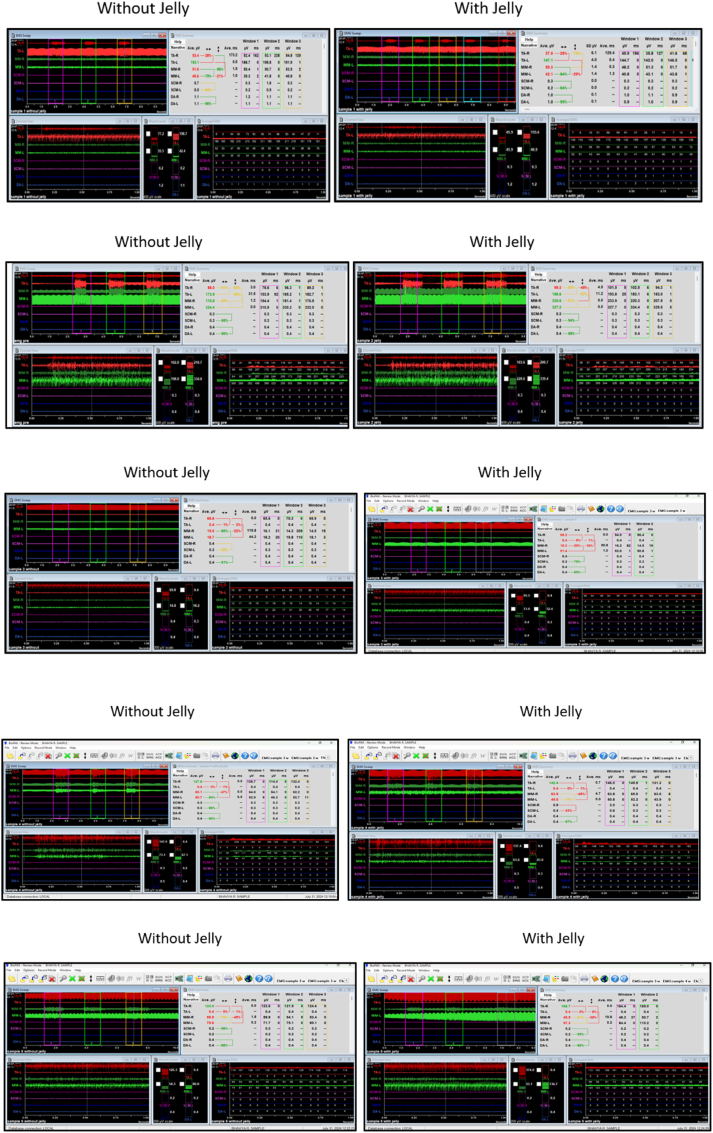
Electromyography of the study participants with and without jelly.

The comprehensive visual representation of the data, highlighting the effects of various jelly-infused taste stimuli on muscle synergy and symmetry. Key measurements, including Synergy Right, Synergy Left, Symmetry Temporalis, and Symmetry Anterior Masseter, are compared across conditions with and without the jelly stimuli. These comparisons showcase how the introduction of different taste stimuli influences muscular coordination (Fig. 3 -Samples 1 to 5).

**Fig. 4:**
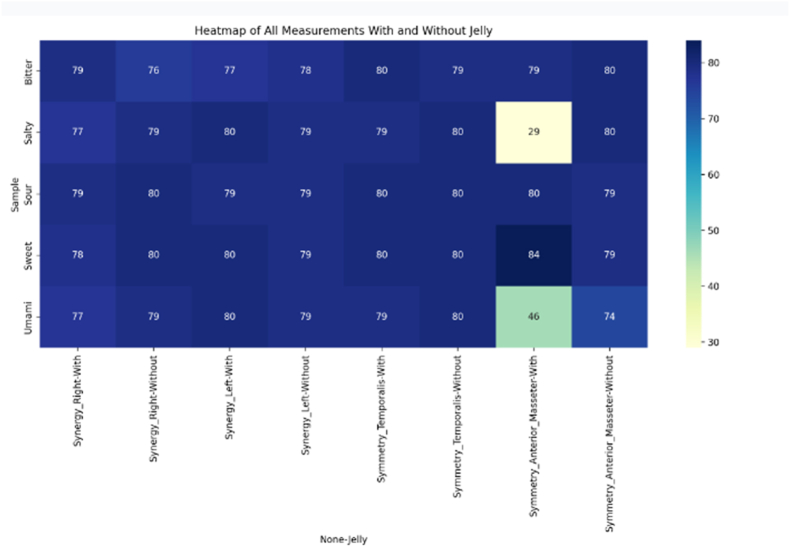
Heatmap of measurements with and without Jelly.

**Fig. 5:**
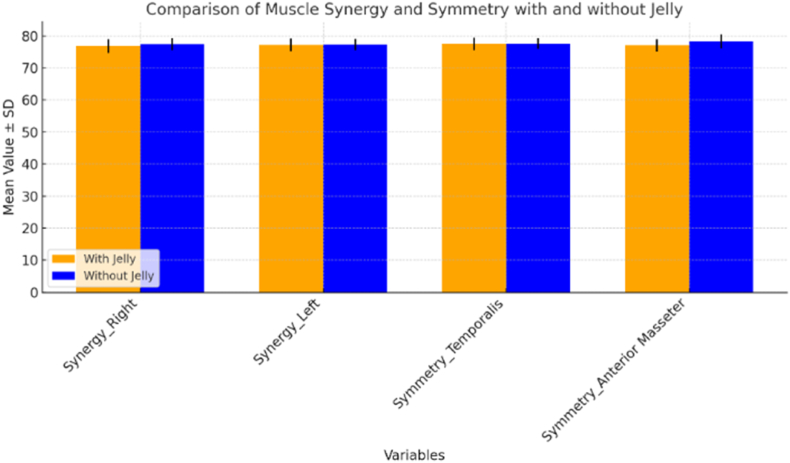
Effects in muscle function with and without jelly.

Additionally, the heatmap offers an at-a-glance overview of these parameters, allowing for easy identification of trends and differences in muscle activity between the right and left sides, as well as between the temporalis and masseter muscles during mastication (Fig. 4).The heatmap values correspond to the mean EMG activity of participants, averaged across trials for each taste condition. This calculation was performed to provide a representative measure of muscle activity patterns across different taste stimuli (see Fig. 5).

The jelly also influences muscle synergy and symmetry in a taste-dependent manner. Synergy Right and Left exhibit subtle fluctuations, with sweet and salty samples showing increased synergy in the presence of jelly, while bitter and umami samples demonstrate reduced synergy. In contrast, Symmetry Temporalis remains relatively stable across all conditions, suggesting that this muscle group is less affected by jelly. Notably, Symmetry Anterior Masseter shows considerable variation, particularly in response to salty and umami stimuli, where symmetry decreases significantly with jelly.

These findings indicate that jelly's effect on muscle function varies according to taste, with certain stimuli eliciting more pronounced changes in muscle activity. The observed variations in synergy and symmetry likely reflect the distinct sensory and motor responses triggered by each taste, underscoring the intricate relationship between gustatory perception and neuromuscular function.

The *t*-test results for Synergy Right, Synergy Left, Symmetry Temporalis, and Symmetry Anterior Masseter all yielded p-values greater than the conventional significance level of 0.05 (0.3796, 0.5346, 0.5447, and 0.2183 respectively). These findings indicate that, when considering all taste samples together, there are no statistically significant differences in muscle synergy or symmetry measurements between the groups with and without jelly. The Symmetry Anterior Masseter showed the lowest p-value (0.2183), suggesting it might be the most sensitive to jelly presence, although still not reaching statistical significance. These results highlight that while our earlier graphical analysis showed some interesting patterns and variations across different taste samples, these differences are not statistically significant when analyzed collectively. This discrepancy between visual patterns and statistical significance underscores the complexity of the relationship between jelly presence and muscle activity across various taste samples, and suggests that more nuanced analyses - such as examining each taste sample separately or employing more sophisticated statistical methods - might be necessary to fully understand the impact of jelly on muscle synergy and symmetry in relation to different tastes. This emphasizes the **lack of statistical significance** in the findings, reinforcing the complexity of the relationship between jelly presence and muscle activity.

## 4 Discussion

The results of this study provide valuable insights into the interaction between gustatory perception and masticatory muscle activity, as influenced by different taste stimuli. The findings suggest that jelly affects muscle synergy and symmetry in a taste-dependent manner, with notable variations observed for certain taste samples. While sweet and salty tastes increased muscle synergy, bitter and umami tastes led to reduced synergy in both the right and left sides of the temporalis and masseter muscles. Interestingly, Symmetry Temporalis showed minimal changes across conditions, implying a degree of stability in temporalis muscle function irrespective of taste stimuli. However, the Symmetry Anterior Masseter demonstrated considerable variation, especially with salty and umami tastes, where symmetry was markedly decreased in the presence of jelly.^11^^,^^12^

Despite these observed trends in the graphical analysis, the statistical analysis, including t-tests, revealed no significant differences in muscle synergy or symmetry between the jelly and non-jelly conditions for all taste stimuli combined. The p-values for Synergy Right (0.379), Synergy Left (0.534), Symmetry Temporalis (0.544), and Symmetry Anterior Masseter (0.218) were all above the 0.05 threshold for statistical significance. Although the Symmetry Anterior Masseter showed the lowest p-value, suggesting a potential sensitivity to jelly presence, this result still did not reach statistical significance, highlighting the need for further investigation.^13^

One potential limitation of this study is the relatively small sample size, which may have reduced the power to detect significant differences. Future studies could benefit from a larger cohort and more sophisticated statistical approaches, such as mixed-effects models, to account for individual variability and the interaction between different tastes and muscle responses. Additionally, examining each taste stimulus independently rather than aggregating them may reveal more specific insights into how distinct gustatory inputs affect masticatory function.

Furthermore, the discrepancy between observed trends in graphical patterns and statistical results points to the complexity of the relationship between taste perception and muscle activity. While previous research suggests that gustatory stimuli can influence neuromuscular responses, the current findings do not provide conclusive evidence to support significant effects of taste on muscle synergy or symmetry.^14^^,^^15^

Future research should address these limitations by increasing the sample size, applying refined statistical techniques, and conducting targeted analyses for individual taste stimuli. Moreover, longitudinal studies or experimental designs incorporating dynamic EMG measurements under controlled taste exposures could enhance our understanding of the nuanced effects of gustatory perception on masticatory muscle function.

By focusing on these methodological refinements and addressing the limitations of the current study, future work can better elucidate the association between taste perception and muscle activity, ultimately contributing to applications in clinical settings and sensory-motor integration research.

## 5 Conclusion

This study contributes to the growing body of research on the sensory-motor integration of taste perception and muscle activity, particularly in the context of mastication. Although no statistically significant differences were found, the trends observed in the graphical analysis suggest a complex and nuanced interaction between taste and muscle function, deserving of further investigation. Continued research in this area could have implications for the management of masticatory disorders, the optimization of chewing efficiency, and the broader understanding of how sensory inputs influence neuromuscular function.

## Patient consent

The patient consent was obtained.

## Ethical clearance

Ethical clearance was obtained from the Institutional Review Board.

## Sources of Funding

Nil.

## Declaration of competing interest

The authors declare that they have no known competing financial interests or personal relationships that could have appeared to influence the work reported in this paper.
